# Denosumab, teriparatide and bisphosphonates for glucocorticoid-induced osteoporosis: a Bayesian network meta-analysis

**DOI:** 10.3389/fphar.2024.1336075

**Published:** 2024-01-19

**Authors:** Liang Dong, Lianghai Jiang, Zhengwei Xu, Xiaobo Zhang

**Affiliations:** ^1^ Department of Orthopedic, Hong-Hui Hospital, Xi’an Jiaotong University College of Medicine, Xi’an, China; ^2^ Department of Spinal Surgery, Qingdao Hospital, University of Health and Rehabilitation Sciences (Qingdao Municipal Hospital), Qingdao, China

**Keywords:** osteoporosis, glucocorticoids, bone mineral density, fracture, network meta-analysis

## Abstract

**Background:** Several medications have been used for glucocorticoids-induced osteoporosis (GIO). However, the best therapeutic option for GIO is still controversial. A Bayesian network meta-analysis was conducted to compare the efficacy and safety of denosumab, teriparatide and bisphosphonates for patients with GIO.

**Methods:** Relevant randomized controlled trials published in PubMed, Embase, Cochrane Library and ClinicalTrials.gov up to August 2023 were searched. The following efficiency and safety outcomes were extracted for comparison: bone mineral density (BMD) percentage changes in lumbar spine, femur neck and total hip, and incidences of adverse events (AEs), serious adverse events (SAEs), vertebrae and non-vertebrae fracture. Bayesian random effects models were used for multiple treatment comparisons.

**Results:** 11 eligible RCTs involving 2,877 patients were identified. All the six medications including alendronate, risedronate, etidronate, zoledronate, teriparatide, and denosumab and were effective in increasing BMD. Teriparatide and denosumab were more effective in improving lumbar spine and femur neck BMD, and reducing vertebrae fracture. Alendronate and denosumab were more effective in improving total hip BMD. Alendronate and teriparatide had the lowest incidences of AEs and SAEs.

**Conclusion:** Teriparatide denosumab and the bisphosphonates are all effective in improving BMD for GIO patients. Based on this network meta-analysis, teriparatide and denosumab have higher efficiency in improving lumbar spine and femur neck BMD, and reducing vertebrae fracture.

**Systematic Review Registration:** 10.17605/OSF.IO/2G8YA, identifier CRD42023456305.

## Introduction

Glucocorticoids are widely used for the treatment of chronic inflammatory and autoimmune diseases, such as rheumatoid arthritis, asthma and systemic lupus erythematosus (Pons-Estel et al., 2018). Long-term use of glucocorticoids can cause a series of side effects, including immune and cardiovascular disorders and osteoporosis (Waljee et al., 2017). Osteoporosis is defined as low bone quality, strength and elevated fracture risk. Primary osteoporosis is due to menopause-related bone demineralization or aging. Secondary osteoporosis is result from pathological conditions or medications other than menopause and aging, which lead to decrease of bone mass and increased fracture risk. Prolonged glucocorticoids use leads to secondary osteoporosis by facilitating osteoclast differentiation and inhibiting osteoblast proliferation, which can result in increased bone resorption (Laan et al., 1993; Wang et al., 2020).

Several treatment options have been proposed for glucocorticoids-induced osteoporosis (GIO). Bisphosphonates including alendronate, risedronate, etidronate and zoledronate are considered as the most common option for GIO (Reid et al., 2009; Buckley et al., 2017; Nasomyont et al., 2021). Numerous studies have proven the efficacy of bisphosphonates for GIO. Bisphosphonates are easily deposited on the surfaces of bone and suppress osteolysis by induction of inhibition and apoptosis of enzymes like farnesyl pyrophosphate synthase in the osteoclasts. However, potential side effects and inconvenient dosing regimens of bisphosphonate may lead to discontinuation of treatment. Other options for GIO include teriparatide and denosumab. Teriparatide is a parathormone analogue that can promote bone formation. Denosumab is a human monoclonal antibody binding to receptor activator of nuclear factor kappa-B ligand and inhibits osteoclastogenesis (Lipton and Goessl, 2011; Body, 2012; Lacey et al., 2012). The best therapeutic option for GIO, however, is still controversial. Several traditional pairwise meta-analysis have been performed to compare different treatments (Yanbeiy and Hansen, 2019; Yamaguchi et al., 2020; Jiang et al., 2022). However, network meta-analysis, having the advantages of indirect comparisons and ranking, is still in lack. Therefore, we conducted a Bayesian network meta-analysis with the aim to compare the efficacy and safety of denosumab, teriparatide and bisphosphonates for patients with GIO.

## Materials and methods

This meta-analysis was performed in accordance with a registered protocol (CRD42023456305).

### Search strategy

This network meta-analysis was performed according to the PRISMA extension statement for network meta-analysis (Hutton et al., 2015). We searched the Pubmed, Embase, Cochrane Library and ClinicalTrials.gov for relevant randomized controlled trials (RCTs) until the end of August 2023. The following search terms were used: osteoporosis, glucocorticoid-induced, steroids, bone mineral density (BMD), bisphosphonate, alendronate, risedronate, etidronate, zoledronate, teriparatide and denosumab. Additionally, references of selected articles were checked for studies that met the criteria.

### Inclusion and exclusion criteria

Two authors screened the relevant studies independently, and disagreements were adjudicated by a third author. Original studies in English were eligible.

The inclusion criteria were: 1) studies that included patients with GIO; 2) double-blind or open-label RCTs lasting at least 12 months; 2) studies focusing on the comparisons among placebo, alendronate, risedronate, etidronate, zoledronate, teriparatide or denosumab; 3) studies reporting at least one of the following outcomes: BMD percentage changes in lumbar spine, total hip and femur neck. Incidences of adverse events (AEs), serious adverse events (SAEs), vertebrae and non-vertebrae fracture.

The exclusion criteria were: 1) duplicate studies for same population with different investigations; 2) studies including patients younger than 18 years old; 3) studies with patients taking medications that may have effect on BMD.

### Data extraction and quality assessment

The following data was extracted from each included RCT by two authors independently: study characteristics (e.g., first author, publication date, location, sample size), antiosteoporosis medications (e.g., type of antiosteoporosis medicine, dosage regimen), patient characteristics (e.g., number of patients, age and sex), percentage changes in BMD, incidence of AEs, SAEs, vertebrae and non-vertebrae fracture.

Quality of the included RCTs were independently evaluated by two authors according to the Cochrane Collaboration’s risk of bias tool for RCTs (Higgins et al., 2011). Disagreements between the two authors were resolved through discussion.

### Data synthesis and analysis

This Bayesian network meta-analysis was conducted by R (version 4.0.3) and gemtc package (van Valkenhoef et al., 2016; Shim et al., 2019). The outcomes of the network meta-analysis were presented by mean difference (MD) and 95% credibility intervals (95% CrI) for continuous data, and by odds ratio (OR) and 95% CrI for dichotomous data. The Markov chain Monte Carlo simulation technique within a Bayesian framework were used to perform the network meta-analysis. Random-effects and consistency models were used in this meta-analysis. The random model used four chains with 10,000 burn-ins and 50,000 iterations. Node-splitting analysis were performed between the direct and indirect evidence for consistency evaluation. Surface under cumulative ranking curve (SUCRA) was calculated to rank the outcomes of each treatment based on a Bayesian approach (Rücker and Schwarzer, 2015). A larger SUCRA value meant a higher rank of the intervention (Salanti et al., 2011; Shim et al., 2019). The publication bias was assessed using the funnel plot and Egger test.

## Results

### Literature search and study characteristics

**FIGURE 1:**
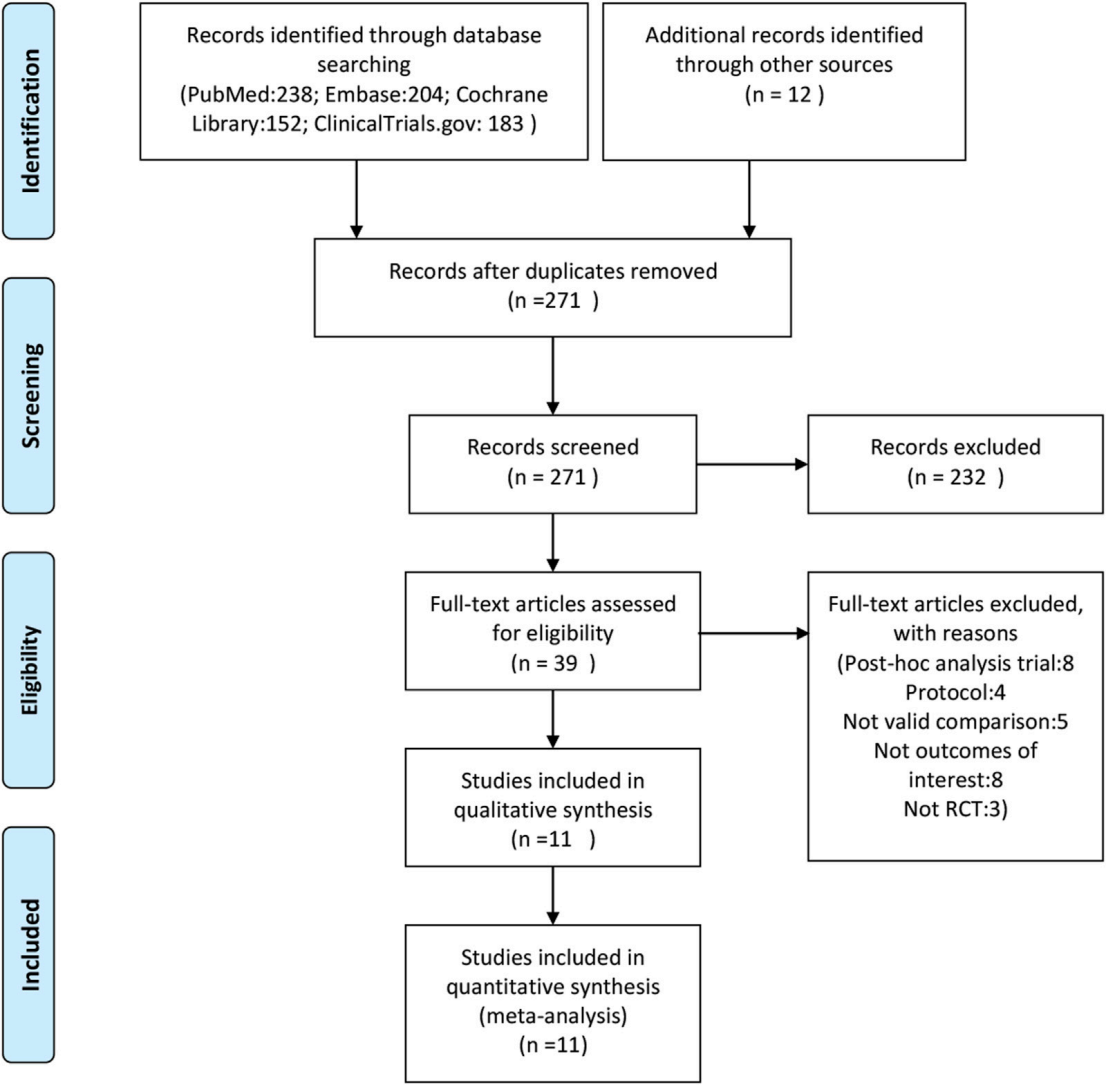
Flow chart of literature search.

**FIGURE 2:**
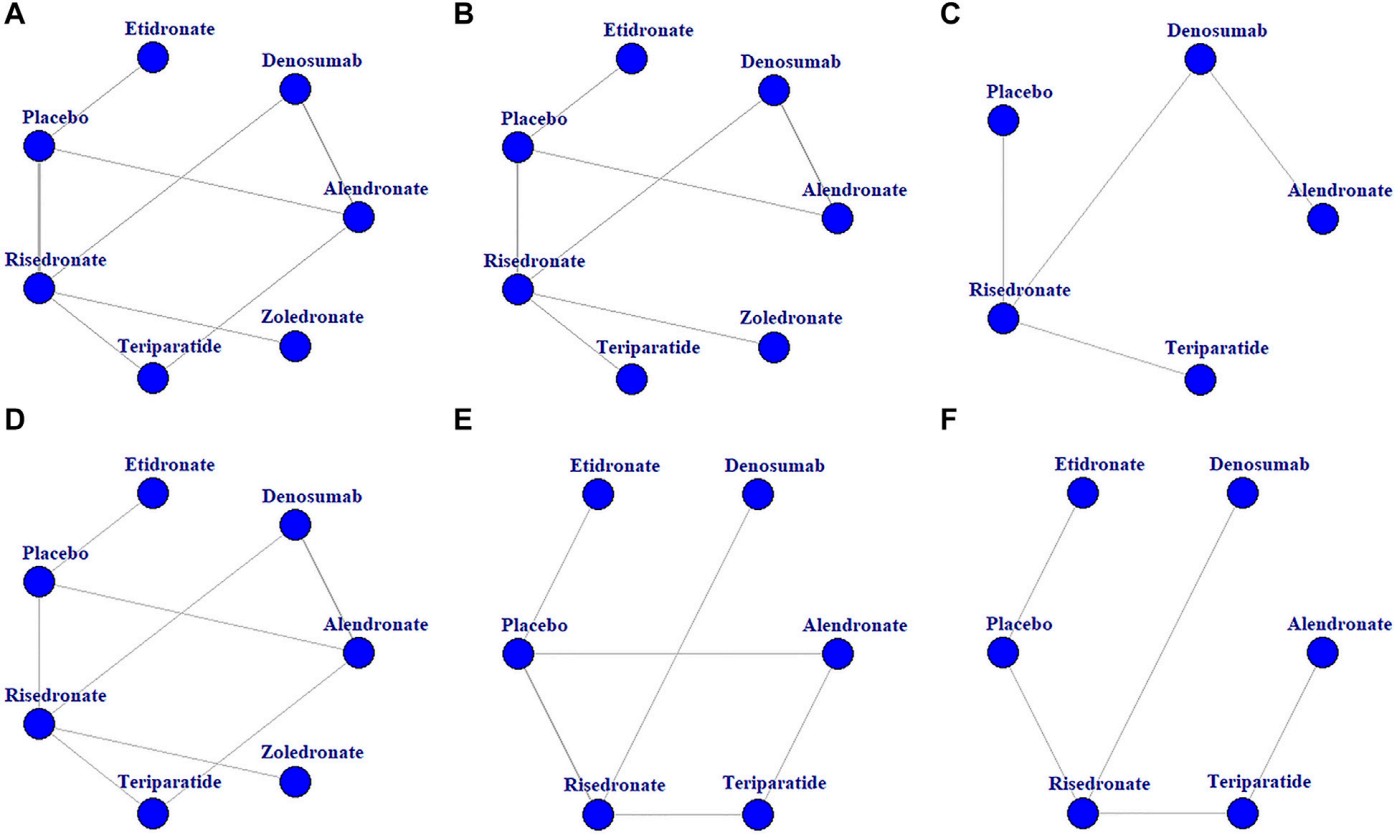
Network diagrams of the comparisons of treatments. BMD percentage changes in lumbar spine **(A)**, femur neck **(B)**, and total hip **(C)**. Incidences of AEs **(D)**, vertebrae fracture **(E)**, and non-vertebrae fracture **(F)**.

A total of 271 potentially relevant articles were identified in the database search. After reviewing the titles, abstracts and full texts, 259 articles were excluded. Eight studies (Langdahl et al., 2009; Losada et al., 2009; Burshell et al., 2010; Devogelaer et al., 2010; Eastell et al., 2010; Sambrook et al., 2012; Farahmand et al., 2013) were excluded for being subgroup analysis or *post hoc* study from previous data. Finally, 11 RCTs (Adachi et al., 1997; Saag et al., 1998; Cohen et al., 1999; Reid et al., 2000; Wallach et al., 2000; Saag et al., 2007; Reid et al., 2009; Glüer et al., 2013; Iseri et al., 2018; Saag et al., 2019; Mok et al., 2021) involving 2,877 patients were included for this meta-analysis. The sample size of the included RCTs ranged from 28 to 545, and the follow-up period ranged from 12 to 52 months. Figure 1 and Figure 2 presents the screening process and the network. Table 1 presents the baseline characteristics of the included RCTs. According to the Cochrane Collaboration’s risk of bias tool for RCTs, final risk of bias of the included studies was low (Supplementary Figure S1).

**TABLE 1:** Baseline characteristics of studies included in this network meta-analysis.

Author, year	Intervention	Sample size	Age (year)	Female/male	Follow-up (months)	Prednisolone-equivalent dose (mg/day)	Steroid duration (months)
Mok et al. (2021)	Denosumab	69	52.0 ± 12.3	68/1	12	5.1 ± 2.9	111 ± 62
	Alendronate	70	48.0 ± 12.9	65/5		5.0 ± 2.4	104 ± 69
Saag et al. (2019)	Denosumab	253	61.5 ± 11.6	185/68	24	12.3 ± 8.09	0–3: 5.1%; 3–12: 32.0%; ≥12: 62.5%
	Risedronate	252	61.3 ± 11.1	185/67		11.1 ± 7.69	0–3: 3.2%; 3–12: 29.8%; ≥12: 66.3%
Iseri et al. (2018)	Denosumab	14	66.5 (39.0–75.8)	6/8	12	5.0 (2.4–8.5)	82.8 (26.4–228)
	Alendronate	14	65.5 (45.0–78.5)	6/8		5.0 (2.5–9.3)	108 (21.6–229.2)
Glüer et al. (2013)	Teriparatide	45	57.5 (12.8)	45/0	18	8.8	85.2
	Risedronate	47	55.1 (15.5)	47/0		8.8	58.8
Reid et al. (2009)	Zoledronate	272	53.2 (14.0)	87/185	12	10.0 (7.5–12.5)	-
	Risedronate	273	52.7 (13.7)	90/183		10.0 (7.5–12.5)	-
Saag et al. (2007)	Teriparatide	214	56.1 ± 13.4	42/172	18	7.5	18
	Alendronate	214	57.3 ± 14.0	41/173		7.8	14.4
Wallach et al. (2000)	Risedronate	174	59.3 (13.20)	63/111	12	-	≤3: 68; 3–6: 9; >6: 97
	Placebo	170	58.0 (13.06)	60/110		-	≤3: 72; 3–6:3; >6: 95
Reid et al. (2000)	Risedronate	100	58 ± 12	36/64	12	15 ± 12	57 ± 58
	Placebo	96	59 ± 12	36/60		15 ± 13	62 ± 72
Cohen et al. (1999)	Risedronate	76	61.9 ± 14.3	27/49	12	20.4 ± 1.9	-
	Placebo	77	57.2 ± 14.7	25/52		21.7 ± 2.0	-
Saag et al. (1998)	Alendronate	157	55 ± 15	44/113	48	10	<4:54; 4–12:31; >12:72
	Placebo	159	54 ± 15	52/107		11	<4:52; 4–12:34; >12:73
Adachi et al. (1997)	Etidronate	67	62 ± 14	28/46	52	21 ± 22	-
	Placebo	74	60 ± 16	26/41		23 ± 22	-

### Efficiency and safety outcomes

**FIGURE 3:**
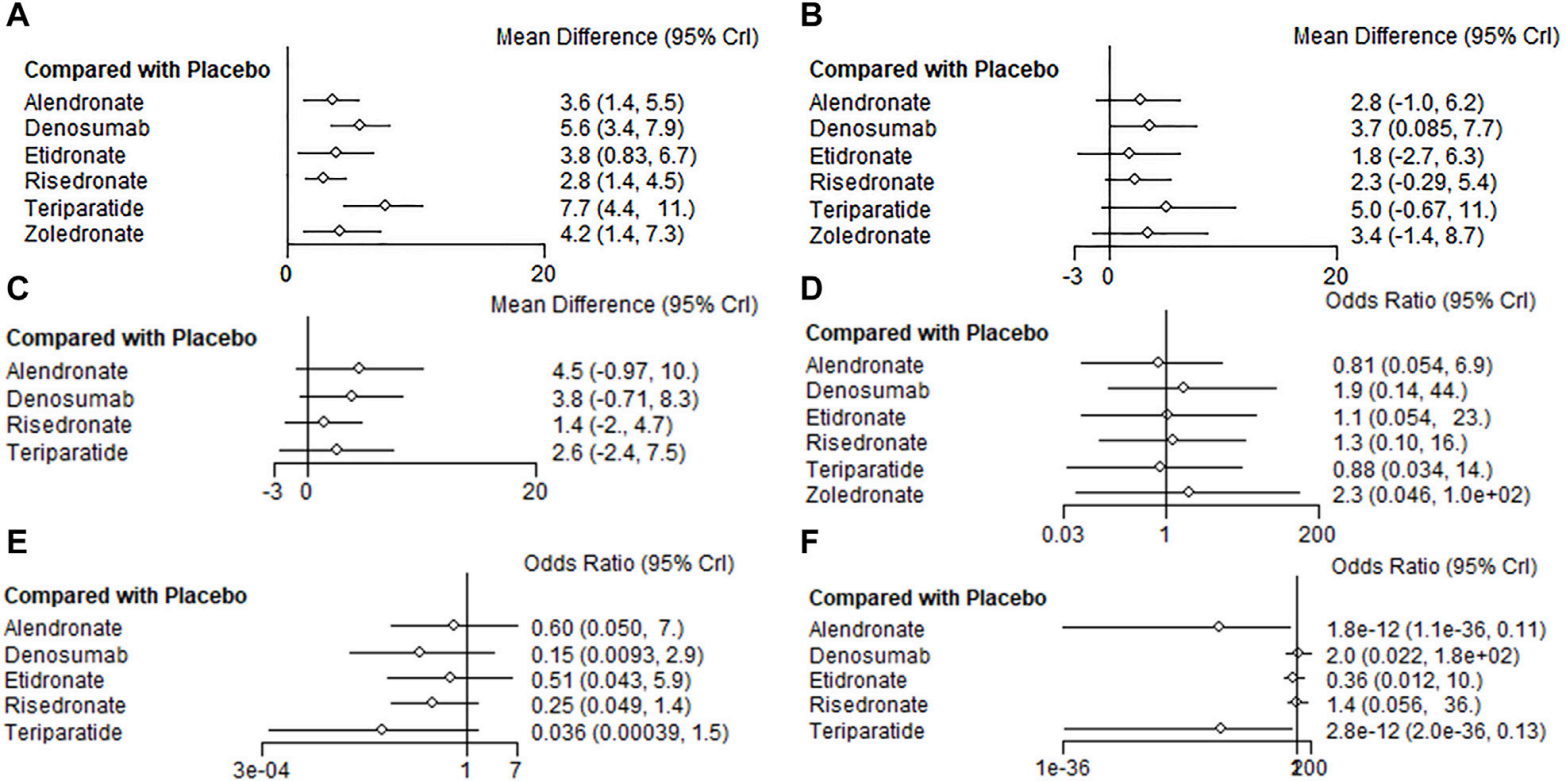
Forest plots of the outcomes. BMD percentage changes in lumbar spine **(A)**, femur neck **(B)**, and total hip **(C)**. Incidences of AEs **(D)**, vertebrae fracture **(E)**, and non-vertebrae fracture **(F)**.

**FIGURE 4:**
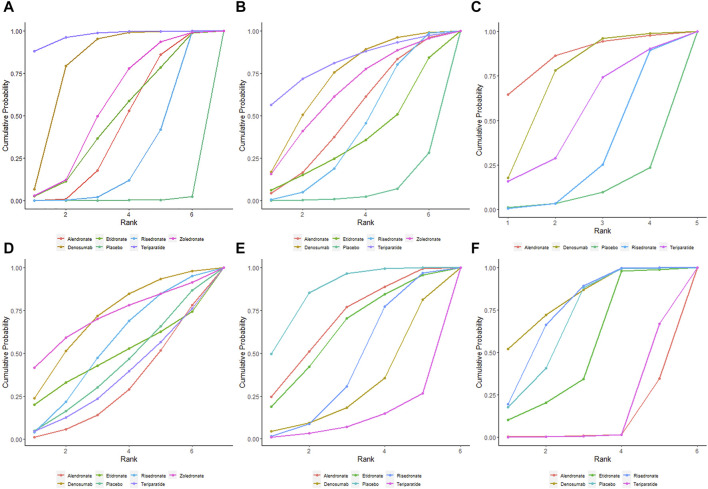
SUCRA indicating the probability ranks of outcomes. BMD percentage changes in lumbar spine **(A)**, femur neck **(B)**, and total hip **(C)**. Incidences of AEs **(D)**, vertebrae fracture **(E)**, and non-vertebrae fracture **(F)**.

At the end of the follow-up period, according to the network meta-analysis, all the six treatments (alendronate, risedronate, etidronate, zoledronate, teriparatide and denosumab) were significantly superior to placebo in improving lumbar spine BMD (*p* < 0.05) (Figure 3A; Table 2A). Based on the SUCRA values of various treatment strategies, teriparatide and denosumab were the best treatments for improving lumbar spine BMD, followed by zoledronate, etidronate, alendronate and risedronate (Figure 4A).

**TABLE 2:** Pooled estimates of the network meta-analysis. BMD percentage changes in lumbar spine (A), femur neck (B), and total hip (C).

A	Placebo	Risedronate	Alendronate	Etidronate	Zoledronate	Denosumab	Teriparatide
Placebo	-	-	-	-	-	-	-
Risedronate	**−2.84 (−4.5, −1.44)**	-	-	-	-	-	-
Alendronate	**−3.64 (−5.55, −1.38)**	−0.8 (−2.61, 1.63)	-	-	-	-	-
Etidronate	**−3.83 (−6.74, −0.83)**	−0.98 (−4.15, 2.5)	−0.2 (−3.92, 3.31)	-	-	-	-
Zoledronate	**−4.17 (−7.3, −1.36)**	−1.36 (−3.9, 1.2)	−0.53 (−4.17, 2.42)	−0.37 (−4.72, 3.64)	-	-	-
Denosumab	**−5.65 (−7.91, −3.39)**	**−2.84 (−4.72, −0.63)**	**−2.01 (−4.08, −0.31)**	−1.82 (−5.59, 1.83)	−1.5 (−4.6, 1.93)	-	-
Teriparatide	**−7.69 (−10.55, −4.43)**	**−4.84 (−7.51, −1.57)**	**−4.06 (−6.6, −1.4)**	−3.86 (−7.94, 0.57)	−3.5 (−7.04, 0.73)	−2.04 (−4.88, 1.22)	-

B	Placebo	Etidronate	Risedronate	Alendronate	Zoledronate	Denosumab	Teriparatide
Placebo	-	-	-	-	-	-	-
Etidronate	−1.79 (−6.27, 2.73)	-	-	-	-	-	-
Risedronate	−2.35 (−5.42, 0.29)	−0.56 (−6.14, 4.54)	-	-	-	-	-
Alendronate	−2.76 (−6.19, 1)	−0.96 (−6.57, 4.93)	−0.41 (−3.88, 3.82)	-	-	-	-
Zoledronate	−3.41 (−8.74, 1.42)	−1.63 (−8.66, 4.88)	−1.09 (−5.29, 3.13)	−0.65 (−6.77, 4.62)	-	-	-
Denosumab	−3.71 (−7.75, −0.09)	−1.92 (−8.07, 3.78)	−1.39 (−4.88, 2.16)	−0.97 (−4.47, 1.87)	−0.32 (−5.77, 5.24)	-	-
Teriparatide	−5.05 (−11.09, 0.67)	−3.26 (−10.89, 3.92)	−2.68 (−7.84, 2.47)	−2.31 (−9.04, 3.82)	−1.6 (−8.15, 5.04)	−1.32 (−7.58, 4.86)	-

C	Placebo	Risedronate	Teriparatide	Denosumab	Alendronate		
Placebo	-	-	-	-	-		
Risedronate	−1.38 (−4.72, 1.96)	-	-	-	-		
Teriparatide	−2.58 (−7.52, 2.45)	−1.18 (−4.86, 2.56)	-	-	-		
Denosumab	−3.8 (−8.3, 0.71)	−2.42 (−5.42, 0.62)	−1.24 (−5.99, 3.49)	-			
Alendronate	−4.5 (−10, 0.97)	−3.11 (−7.46, 1.24)	−1.94 (−7.65, 3.76)	−0.7 (−3.83, 2.42)	-		

Note: The column treatments were compared with the row treatments. The BMD, percentage changes were presented by MD, and 95% CrI. MDs, with a Bayesian *p*-value of less than 0.05 were in bold.

Based on the network meta-analysis, all the six treatments had higher percentage changes in femur neck BMD than the placebo (Figure 3B; Table 2A). According to the SUCRA values, teriparatide and denosumab scored the highest, followed by zoledronate, alendronate, risedronate and etidronate (Figure 4B).

Concerning total hip BMD, four treatments were included in comparison. All the five treatments had higher BMD percentage changes than the placebo but without significance (*p* > 0.05) (Figure 3C; Table 2B). Regarding to SUCRA ranking analysis, alendronate and denosumab ranked highest, followed by teriparatide and risedronate (Figure 4C).

Regarding to the incidence of AEs, alendronate and teriparatide had the lowest incidence of AEs, followed by etidronate, risedronate, denosumab and zoledronate (Figure 4D). Teriparatide and alendronate had the lowest incidence of SAEs, followed by zoledronate, risedronate and denosumab.

With respect to the incidences of vertebrae fracture, teriparatide and denosumab had the lowest incidence of vertebrae fracture, followed by risedronate, etidronate and alendronate (Figure 4E). Alendronate and teriparatide had the lowest incidence of non-vertebrae fracture, followed by etidronate, risedronate and denosumab (Figure 4F).

Node-splitting analysis showed no significant heterogeneity or inconsistency occurred in direct and indirect evidence (*p* > 0.05) (Supplementary Figure S2). For comparisons with more than ten studies included, funnel plots and Egger’s test were used to assess publication bias. Regarding to comparisons of percentage changes in lumbar spine BMD, the funnel plots looked symmetric, and the Egger’s test showed no significant potential publication bias (*p* > 0.05) (Supplementary Figure S3).

## Discussion

This present Bayesian network meta-analysis summarized the latest information and provided a ranking of various treatments (alendronate, risedronate, etidronate, zoledronate, teriparatide and denosumab) in treating GIO. A previous meta-analysis by Yuan et al. (2023) compared denosumab, teriparatide, and oral bisphosphonates (alendronate and risedronate) in the prevention of GIO, and noted that teriparatide and denosumab were similar or even superior to bisphosphonates. There are some differences between the meta-analysis by Yuan et al. (2023) and our study. Firstly, unlike the pairwise meta-analysis by Yuan et al. (2023), we conducted a Bayesian network meta-analysis. Network meta-analysis was performed to compensate for the lack of face-to-face comparisons among various treatments. Network meta-analysis has the advantages of indirect comparisons and providing ranking of different treatments. Secondly, in the meta-analysis by Yuan et al. (2023), bisphosphonates including alendronate and risedronate were compared with denosumab and teriparatide as a whole, while alendronate and risedronate, in fact, are different to some extent. Besides, the comparisons between denosumab and teriparatide were not performed in the meta-analysis by Yuan et al. (2023). Oppositely, in our network meta-analysis, alendronate, risedronate, etidronate, zoledronate, denosumab and teriparatide were compared with each other separately. Table 2 showed the comparison results of BMD percentage changes between every two treatments in detail.

To our knowledge, there is only one network meta-analysis performed by Migliorini et al. on antiresorptive treatments for GIO (Migliorini et al., 2022). In this network meta-analysis, the treatments included alendronate, zoledronate, risedronate, denosumab and etidronate, but not teriparatide. Teriparatide has the effect of promoting bone formation. Teriparatide has been proved effective in treating GIO and has been widely used (Solomon et al., 2017; Kaneko et al., 2018), so we conducted the present network meta-analysis including denosumab, teriparatide and bisphosphonates. Our study provided suggestions to help professionals and GIO patients make decisions on their treatment.

In the present network meta-analysis, teriparatide and denosumab ranked the first two in improving lumbar spine BMD, followed by the bisphosphonates. This result was consistent with previous RCTs. The study by Glüer et al. and Saag et al. suggested that teriparatide was more effective in increasing the BMD than the bisphosphonates (Saag et al., 2007; Glüer et al., 2013). And numerous studies have proved denosumab was superior in improving lumbar spine BMD than the bisphosphonates (Iseri et al., 2018; Saag et al., 2019; Mok et al., 2021). Similarly, in the meta-analysis by Yuan et al. (2023), teriparatide and denosumab were shown to be superior in improving BMD in lumbar spine than the bisphosphonates. In the network meta-analysis by Migliorini et al. (2022), denosumab and alendronate ranked the first two in improving lumbar spine BMD. However, teriparatide was not included in the study, whose limitation was compensated for by our study.

Based on the present network meta-analysis, teriparatide and denosumab ranked the first two in improving femur neck BMD, while alendronate and denosumab ranked the first two in improving total hip BMD. This difference in BMD improvement at various skeletal sites may be associated with different proportion of trabecular bone and different treatment-related changes in bone density and architecture (Choksi et al., 2018). Anti-resorptive medications (denosumab and bisphosphonates) decrease the endosteal diameter by increasing endosteal bone volume, but do not expand periosteal bone. On the other hand, anabolic agent (teriparatide) leads to increase in periosteal bone and increase in endosteal bone resorption simultaneously, which results in a bone without much cortical thickness change (Choksi et al., 2018). In the meta-analysis by Yuan et al. (2023), teriparatide was superior to bisphosphonates in increasing hip BMD. However, our network meta-analysis showed no significant differences between teriparatide and the bisphosphonates (Table 2), and alendronate ranked the first in increasing hip BMD (Figure 4). This result was consistent with the network by Migliorini et al. (2022), which noted alendronate was superior to other treatments. Further studies are needed to explore the efficacy of increasing hip BMD in various treatments.

With respect to the safety of treatments, alendronate and teriparatide had the lowest incidences of AEs and SAEs. Similarly, the network meta-analysis by Migliorini et al. noted the alendronate group had the lowest SAEs (Migliorini et al., 2022). Regarding to reducing the incidence of vertebrae fracture, teriparatide and denosumab ranked the first two, which was consistent with the result of lumbar spine BMD improvement.

There are several limitations in our study. First, patients included in the network meta-analysis had various backgrounds such as indications for glucocorticoid administration and the duration of therapy, which may lead to significant heterogeneity. Second, length of the follow-up varied among included studies, which may result in potential heterogeneity. Most of the included studies had a follow-up time of 12–24 months, while two studies (Adachi et al., 1997; Saag et al., 1998) had longer follow-up time of 48 and 52 months respectively. Third, the number of included studies and the sample size are relatively small.

## Conclusion

For patients with GIO, teriparatide, denosumab and bisphosphonates are all effective in improving BMD. Based on this network meta-analysis, teriparatide and denosumab were superior in improving BMD in lumbar spine and femur neck, and reducing vertebrae fracture.

## Data Availability

The original contributions presented in the study are included in the article/Supplementary Material, further inquiries can be directed to the corresponding author.
