# Supplementary figures and images for: Denosumab, teriparatide and bisphosphonates for glucocorticoid-induced osteoporosis: a Bayesian network meta-analysis
Source: Front Pharmacol. 2024 Jan 19;15:1336075. doi: 10.3389/fphar.2024.1336075 (PMC10834754; doi:10.3389/fphar.2024.1336075)

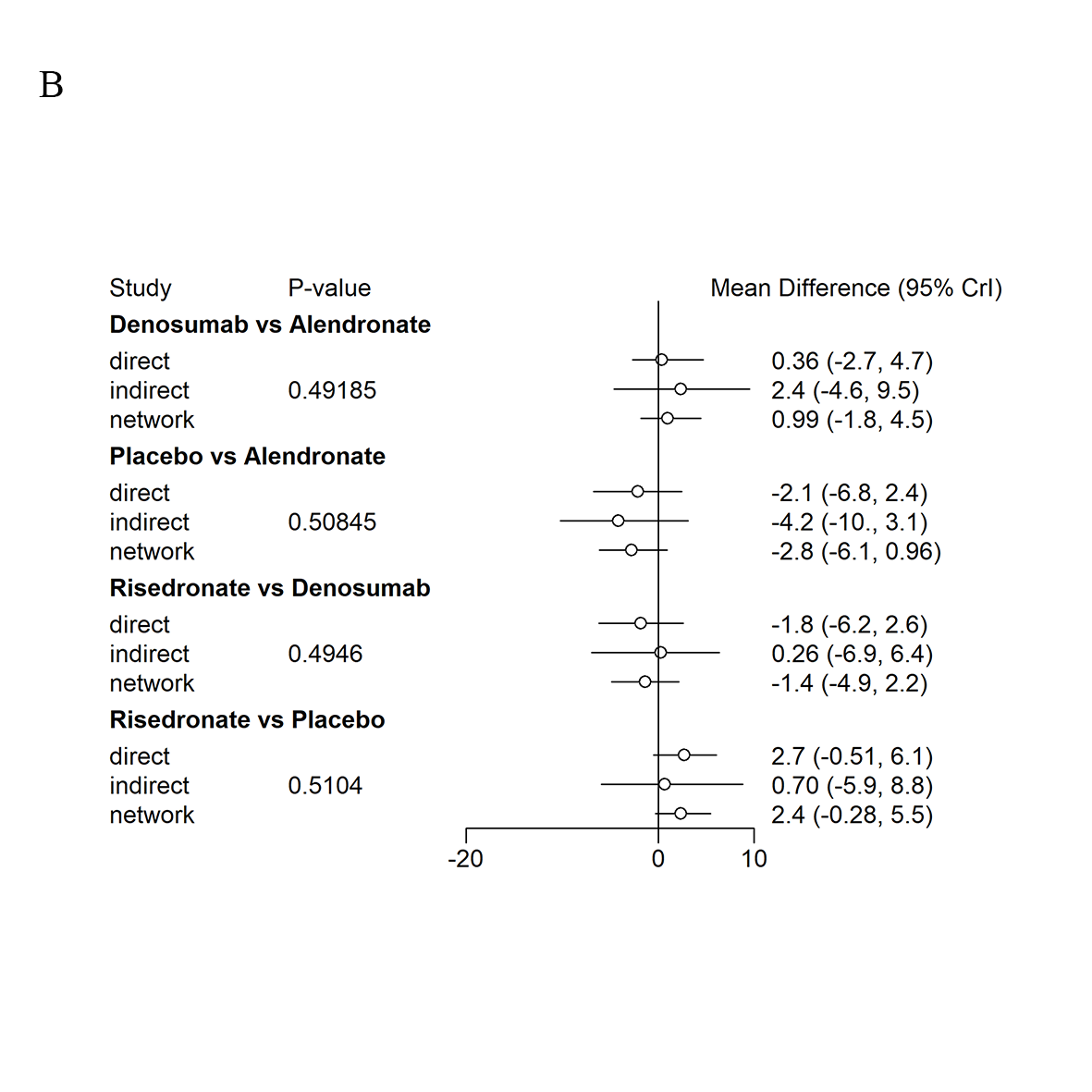

Supplement: Supplementary file 1 [file Image3.TIFF]

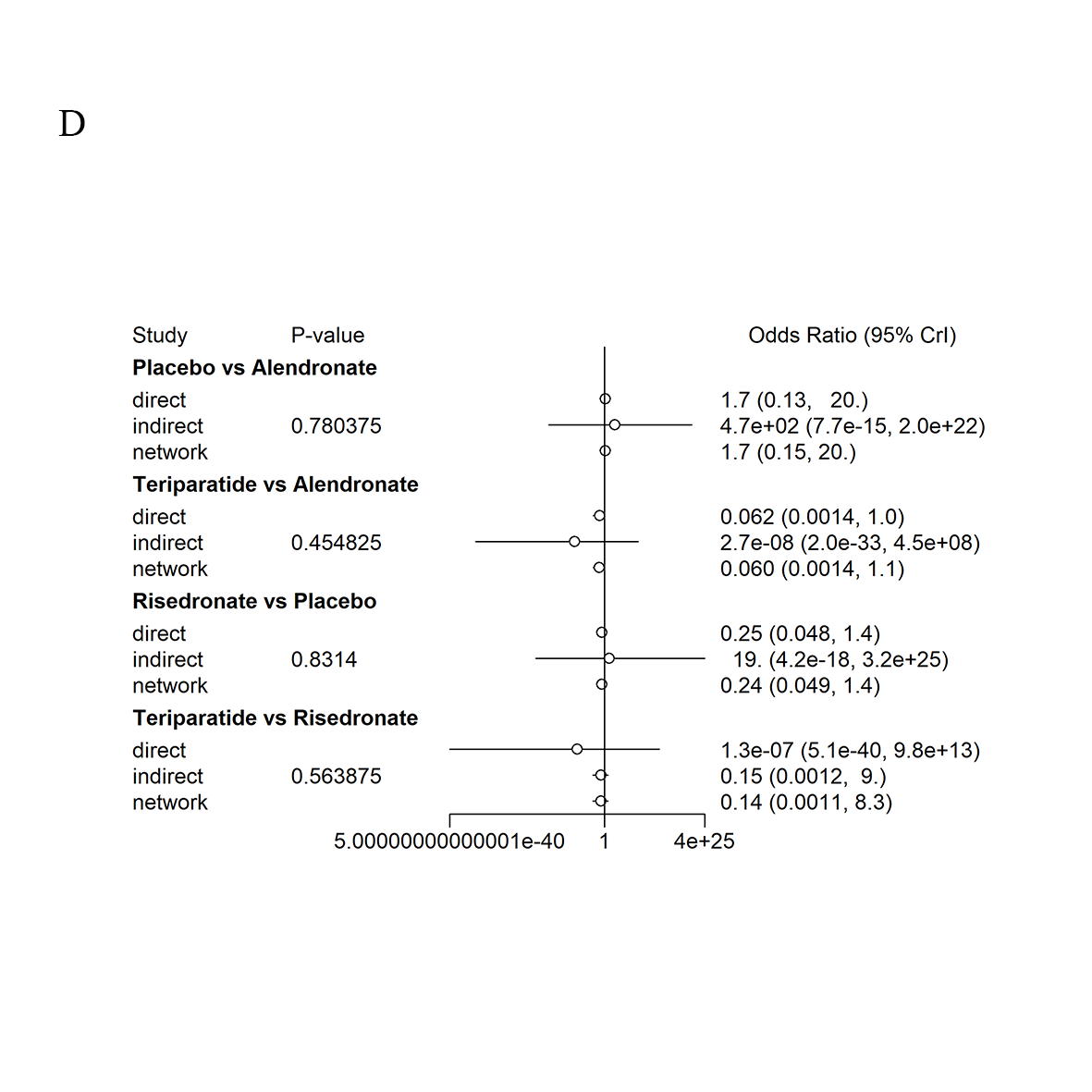

Supplement: Supplementary file 3 [file Image5.TIFF]

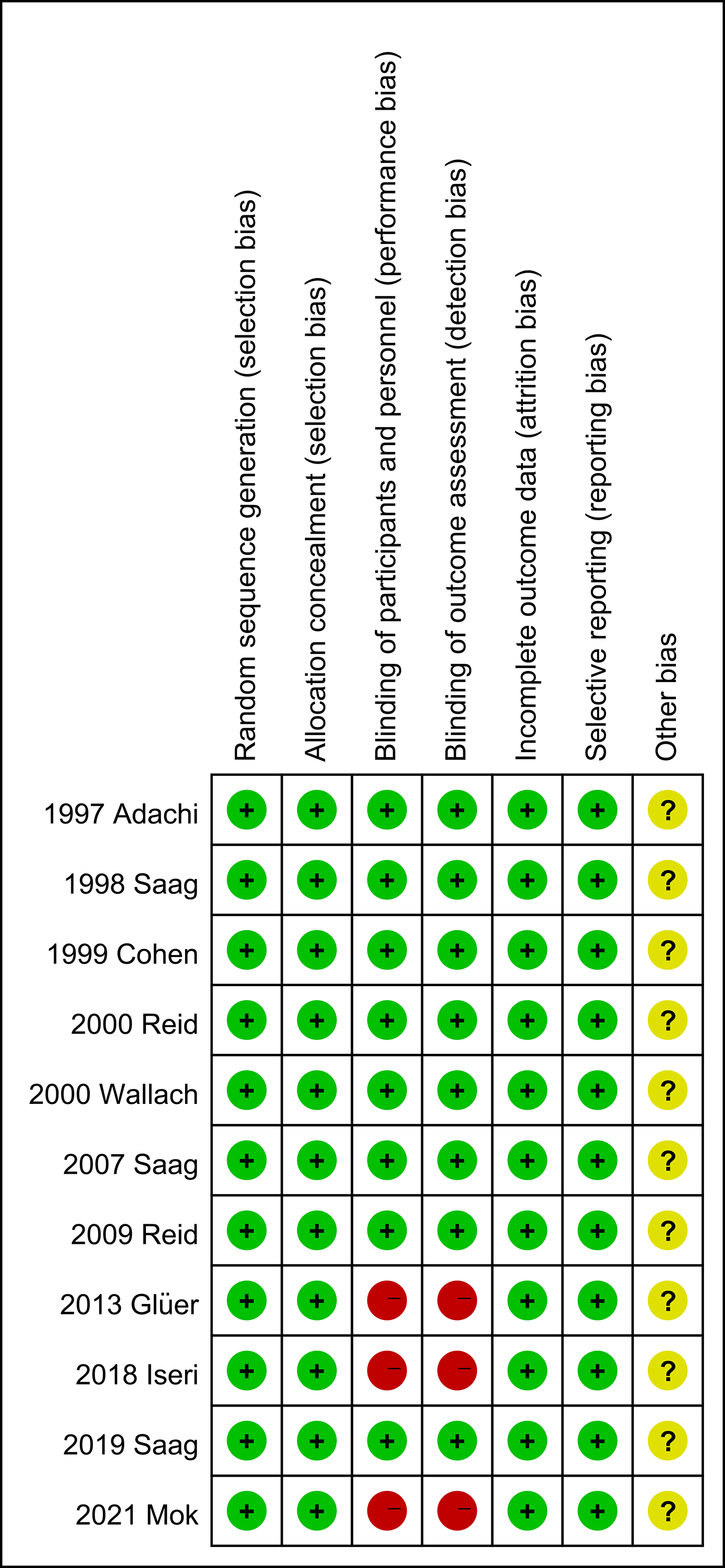

Supplement: Supplementary file 4 [file Image1.TIF]

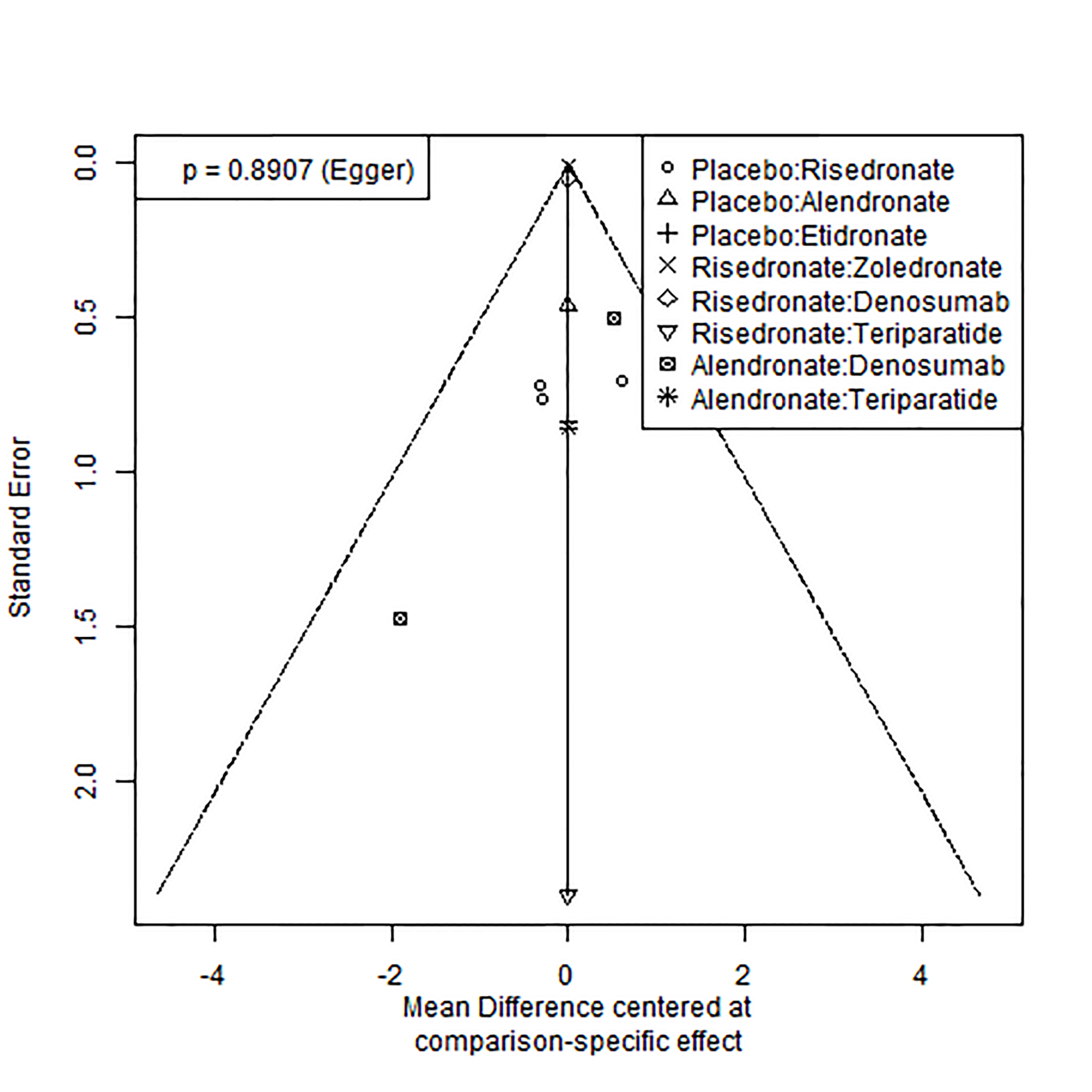

Supplement: Supplementary file 6 [file Image6.TIFF]

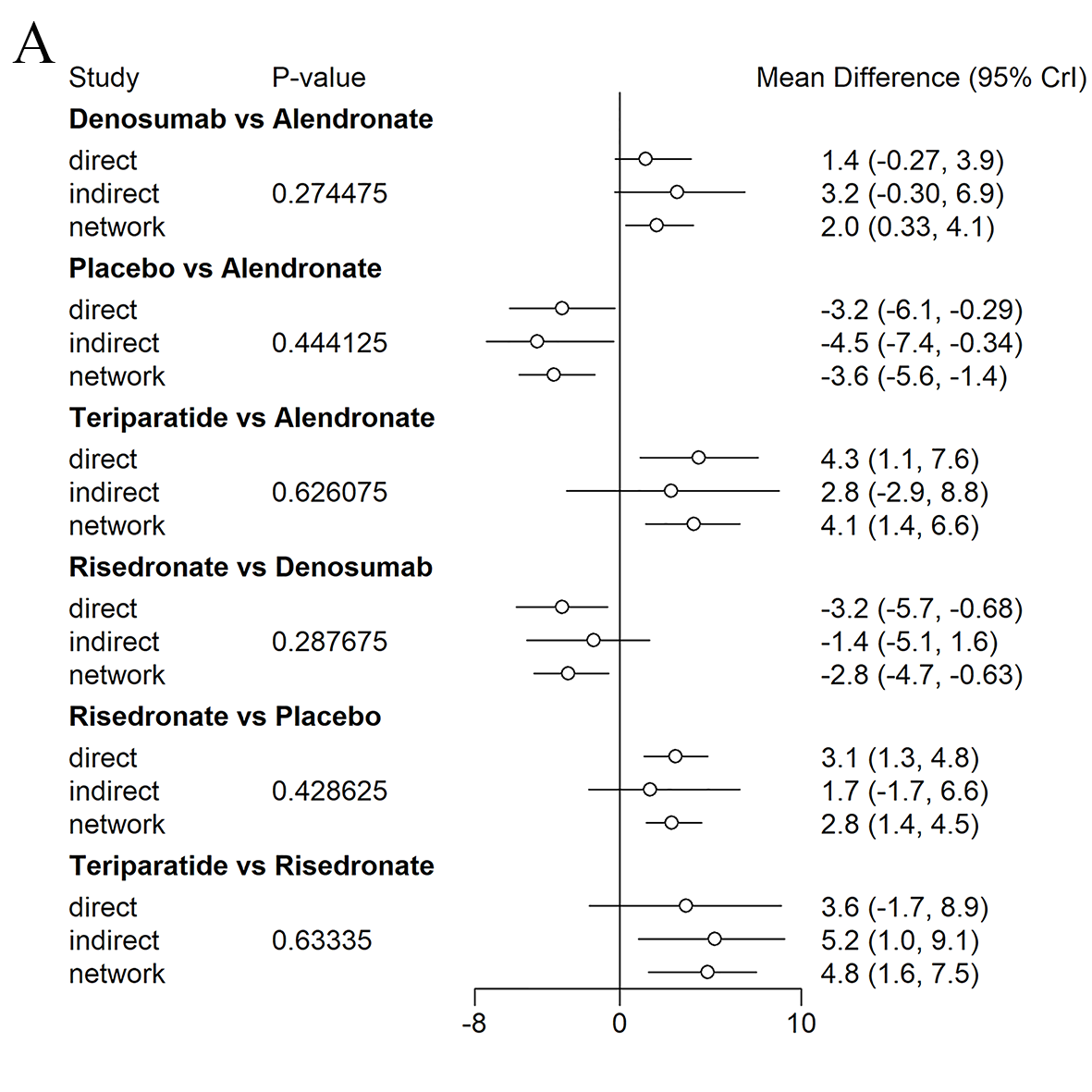

Supplement: Supplementary file 7 [file Image2.TIFF]

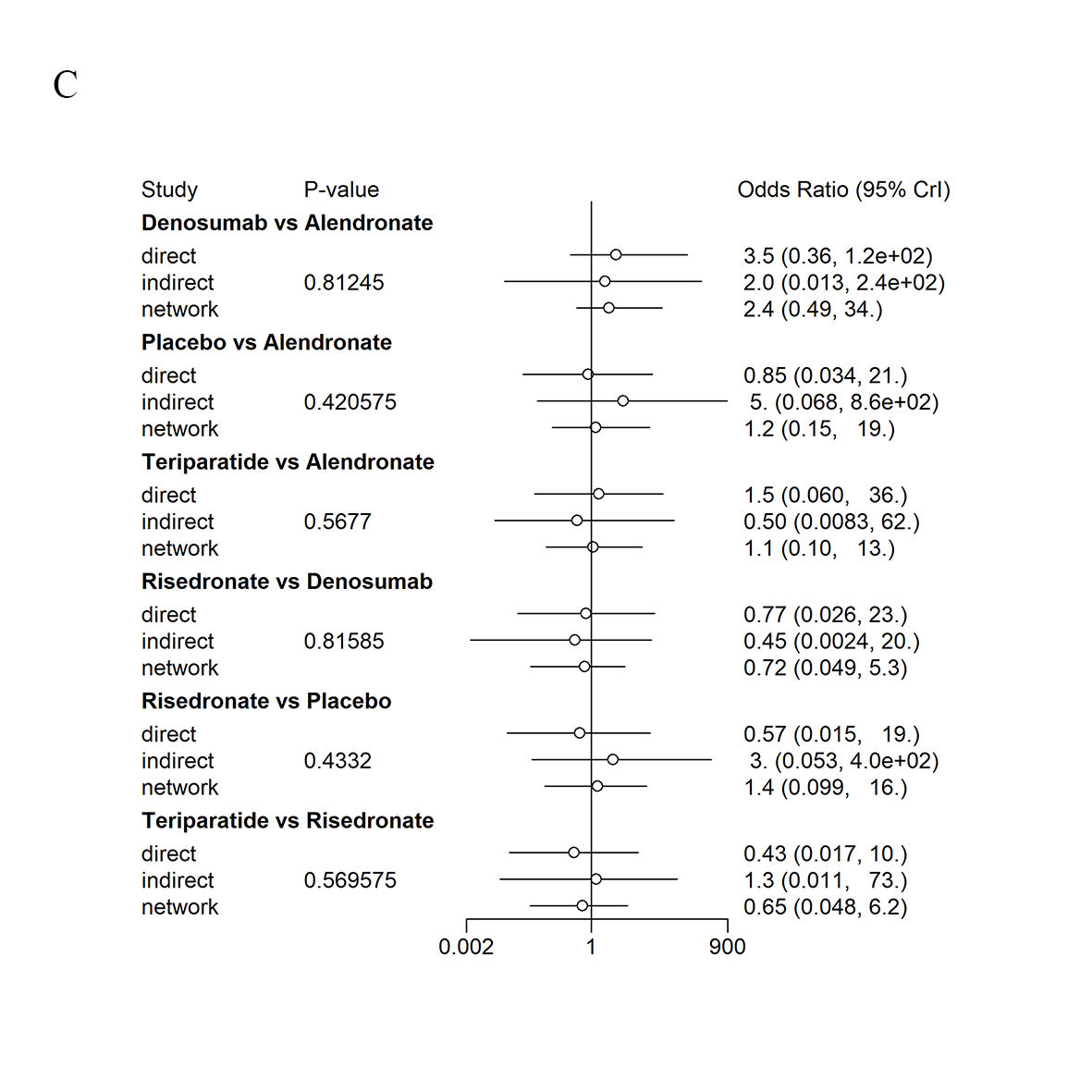

Supplement: Supplementary file 8 [file Image4.TIFF]
