# Supplementary material for: Denosumab, teriparatide and bisphosphonates for glucocorticoid-induced osteoporosis: a Bayesian network meta-analysis
Source: Front Pharmacol. 2024 Jan 19;15:1336075. doi: 10.3389/fphar.2024.1336075 (PMC10834754; doi:10.3389/fphar.2024.1336075)
Supplement: Supplementary file 2 [file Table1.DOCX]

**Supplementary Figure 1.** Quality assessment of the 11 studies included.

**Supplementary Figure 2.** Node-splitting analysis showed no significant inconsistency occurred in direct and indirect evidence. BMD percentage changes in lumbar spine (A) and femur neck (B). Incidences of AEs (C) and vertebrae fracture (D).

**Supplementary Figure 3.** Funnel plot of the studies in the analysis of lumbar spine BMD percentage changes.
